# Experimental evidence for the ancestry of allotetraploid *Trifolium repens* and creation of synthetic forms with value for plant breeding

**DOI:** 10.1186/1471-2229-12-55

**Published:** 2012-04-24

**Authors:** Warren M Williams, Nicholas W Ellison, Helal A Ansari, Isabelle M Verry, S Wajid Hussain

**Affiliations:** 1 AgResearch Grasslands Research Centre, Private Bag 11008, Palmerston North, 4442, New Zealand; 2 College of Sciences, Massey University, Palmerston North, 4442, New Zealand

**Keywords:** *Trifolium repens*, White clover, Allopolyploid, Interspecific hybridization

## Abstract

**Background:**

White clover (*Trifolium repens*) is a ubiquitous weed of the temperate world that through use of improved cultivars has also become the most important legume of grazed pastures world-wide. It has long been suspected to be allotetraploid, but the diploid ancestral species have remained elusive. Putative diploid ancestors were indicated by DNA sequence phylogeny to be *T. pallescens* and *T. occidentale*. Here, we use further DNA evidence as well as a combination of molecular cytogenetics (FISH and GISH) and experimental hybridization to test the hypothesis that white clover originated as a hybrid between *T. pallescens* and *T. occidentale*.

**Results:**

*T. pallescens* plants were identified with chloroplast *trnL* intron DNA sequences identical to those of white clover. Similarly, *T. occidentale* plants with nuclear ITS sequences identical to white clover were also identified. Reciprocal GISH experiments, alternately using labeled genomic DNA probes from each of the putative ancestral species on the same white clover cells, showed that half of the chromosomes hybridized with each probe. F_1_ hybrids were generated by embryo rescue and these showed strong interspecific chromosome pairing and produced a significant frequency of unreduced gametes, indicating the likely mode of polyploidization. The F_1_ hybrids are inter-fertile with white clover and function as synthetic white clovers, a valuable new resource for the re-incorporation of ancestral genomes into modern white clover for future plant breeding.

**Conclusions:**

Evidence from DNA sequence analyses, molecular cytogenetics, interspecific hybridization and breeding experiments supports the hypothesis that a diploid alpine species (*T. pallescens*) hybridized with a diploid coastal species (*T. occidentale*) to generate tetraploid *T. repens*. The coming together of these two narrowly adapted species (one alpine and the other maritime), along with allotetraploidy, has led to a transgressive hybrid with a broad adaptive range.

## Background

White clover, an allotetraploid (2n=4x=32) stoloniferous herb, is naturally distributed through the grasslands of Europe, W Asia and N Africa, from low to high latitudes and altitudes and, because of its broad adaptation, has become the most extensively used legume of grazed pasture world-wide. Its origin has been long debated [1-4]. The identity of the ancestors has remained elusive and, despite many attempts [e.g. [3,5-7] there has been no successful re-synthesis. A phylogenetic analysis of *Trifolium* based on the nuclear internal transcribed spacer region of 18 S–26S rDNA (ITS) and chloroplast *trnL* intron DNA (cpDNA) sequences [8] suggested that the closest extant diploid ancestors were *T. pallescens* (2n=2x=16) and *T. occidentale* (2n=2x=16)*.*

**Figure 1:**
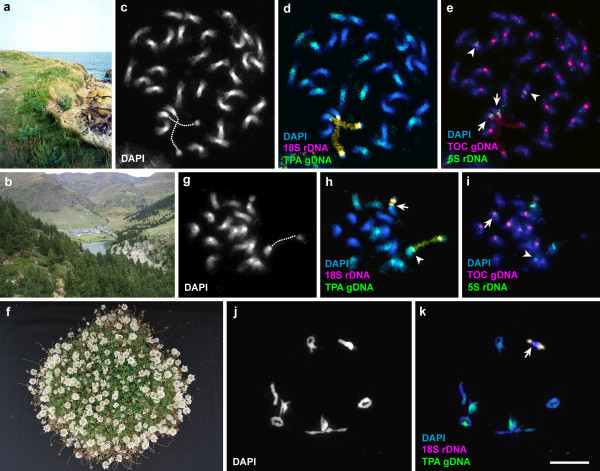
**Molecular cytogenetic and experimental breeding evidence for the ancestry of white clover from a hybrid between two species with contrasting habitats. (a, b)** Habitat surroundings of *T. occidentale* (sea-level, SE Ireland) and *T. pallescens* (2,000m, Pyrenees, NE Spain), respectively. **(c–e)** Reciprocal GISH-FISH on an early metaphase cell from *T. repens*. **(c)** DAPI stained cell in grey-scale. Dotted guide-lines represent decondensed NORs. **(d)** GISH-FISH on the same cell as in **c** using genomic DNA of *T. pallescens* (*green*) and 18S rDNA (*red*). **(e)**Reprobing of the same cell as in **d** with genomic DNA of *T. occidentale* (*red*) and 5 S rDNA (*green*). *Arrows* indicate a *T. occidentale*-derived NOR chromosome pair and *arrowheads* a *T. pallescens*-derived chromosome pair with 5 S rDNA signals. **(f)** F_1_ hybrid plant 440–1. **(gi)** An early somatic metaphase cell from hybrid 440–1 subjected to reciprocal GISH-FISH. **(g)** DAPI stained cell in grey-scale. Dotted guideline represents a decondensed NOR.**(h)** GISH-FISH on the same cell as in **g** using genomic DNA of *T. pallescens* (*green*) and 18S rDNA (*red*). *Arrow* and *arrowhead* indicate *T. occidentale*- and *T. pallescens*-derived NOR chromosomes, respectively. **(i)** Reprobing of the same cell as in **h** with genomic DNA of *T. occidentale* (*red*) and 5S rDNA (*green*). *Arrow* and *arrowhead* indicate *T. occidentale*- and *T. pallescens*-derived chromosomes with 5S rDNA signals, respectively. **(j–k)** A meiotic metaphase cell from hybrid 440–1 subjected to GISH-FISH. **(j)** DAPI stained meiotic metaphase I cell in grey scale showing eight bivalents. **(k)** GISH-FISH on the same cell as in **j** using genomic DNA of *T. pallescens* (*green*) and 18S rDNA (*red*). Bivalent formation involving NOR chromosomes (*arrow*) derived from two parental species and differential hybridization of *T. pallescens* DNA indicate homoeologous pairing. Yellow colour of NORs in **d**, **h**, and **k** is due to simultaneous hybridization of *T. pallescens* DNA (*green*) and 18S rDNA (*red*). Bar **(k)** represents 10m and applies to all chromosomal micrographs.

*T. occidentale* is a predominantly self-fertile, strictly maritime species with a very narrow adaptation, occurring only very close to the sea in confined habitats on the gulf-stream coasts of Europe [9] (Figure 1a). *T. pallescens* is a predominantly cross-pollinating but self-fertile alpine clover, presently occurring only above 1,800 meters in Europe (Figure 1b). It has a narrow adaptation within the alpine zone [10].

Nearly identical ITS sequences [8], SNP analyses [11] and synteny of SSR markers [12] strongly supported *T. occidentale* as the source of one sub-genome. Chloroplast DNA sequences of *T. pallescens* were similar to white clover, while all 200 other species investigated were markedly more divergent [8]. To-date, based on cpDNA, *T. pallescens* is the closest extant species to the maternal ancestor of white clover. Nevertheless, genic SNP comparisons of *T. pallescens* and white clover sub-genomes showed weaker than expected matching, and it was suggested that a taxon closely related to *T. pallescens*, but as yet unidentified, may have been the ancestor [11].

We undertook experimental tests of the hypothesis that white clover arose following hybridization between *T. pallescens* and *T. occidentale*, and here present evidence from DNA sequence analyses, molecular cytogenetics, interspecific hybridization and post-hybridization experiments. The results support the hypothesis and suggest a proposed sequence of events that led to the origin of this widely adapted polyploid species from two very narrowly adapted diploid ancestors via unreduced gametes in an otherwise sterile interspecific F_1_ hybrid. The ability to produce synthetic forms has opened the way to expand the gene-pool of white clover to include current populations of the ancestral species.

## Results

### DNA sequences

In the present work, comparisons were made of the cpDNA sequences of white clover and five geographically divergent *T. pallescens* accessions, all verified by their distinctive ITS DNA sequence. Two *T. pallescens* specimens from N Greece had identical cpDNA sequences (GenBank JN881726, JN881727) to the 580bp*T. repens* reference sequence (GenBank DQ311961). An Austrian (Tyrol) population AZ4856 and AZ1895 (source uncertain) differed from *T. repens* only at positions 143–148, where AAAAAA in *T. repens* was reduced to AA in *T. pallescens*[8]. A Spanish Pyrenees population (AZ4837, GenBank JN982466) was identical to *T. repens* at that site but differed by a five bp ATATA insertion at positions 289–290. Analysis of ITS sequences from several *T. occidentale* populations supported the hypothesis that *T. occidentale* was the other ancestral parent. Some *T. occidentale* populations had identical 738bp ITS sequences to white clover, while others differed from white clover at one of two SNP positions.

### Genomic *in situ* hybridization

Genomic *in situ* hybridization (GISH) using labeled *T. pallescens* genomic DNA showed that half of the chromosomes of white clover hybridized strongly (Figure 1c, d), supporting the hypothesis that *T. pallescens*, or a very close relative, was an ancestor. The arms of the single pair of *T. repens* NOR-carrying chromosomes did not show hybridization, indicating that this chromosome pair was contributed by the other ancestral parent. A reciprocal GISH experiment, using labeled genomic DNA of *T. occidentale* on the same preparations (Figure 1e), led to the stronger hybridization of centromeric regions and faint arms of the other 16 chromosomes, including the two NOR-carrying chromosomes, and supporting *T. occidentale* as the donor of the NOR-bearing genome. Stronger GISH hybridization in the centromeric regions can be caused by the clustering of repetitive sequences in this region [13]. Major clustering of a repetitive sequence, TrR350, at centromeres of *T. repens* and *T. occidentale* has been reported previously [14]. It may also be noted that a pair of chromosomes marked only with a minor 5S rDNA sequence (Figure 1e) is strongly hybridized with *T. pallescens* genomic DNA (Figure 1d).

### Hybridization of *T. pallescens* and *T. occidentale*

No mature seeds were recovered from reciprocal interspecific crosses and no hybrid embryos were formed when *T. occidentale* was used as the female parent. Pollination of *T. pallescens* with *T. occidentale* yielded frequent 0.5mm-long torpedo-stage embryos that were rescued nine days post-pollination and cultured on the artificial media where they developed into plantlets. Approximately 200 plantlets from 18 crosses were transplanted to potting mix in the greenhouse, where most languished, failed to develop beyond 3–5 leaves and did not flower. Only four developed into large plants that grew strongly and flowered prolifically (Figure 1f). All four hybrids were derived from AZ1895 *T. pallescens* as female and two of the four had the same parents (Table 1).

**Table 1:** **Parents and leaf marks of F**_**1**_***T. pallescens*x*T. occidentale*hybrids that reached maturity**

**F**_**1**_** plant**	***T. pallescens* female**	***T. occidentale* male**	**Leaf mark**
440–1	AZ 18953 self-2	OCD 1166–4	Unmarked
1854	AZ 1895–18	OCD 1168–14	Unmarked
Hybrid 3	AZ 1895–3 self-2 self-49	OCD 1162–17	Unmarked
Hybrid 4	AZ 1895–3 self-2 self-49	OCD 1162–17	V mark

Small leaflets densely covered the hybrid plants. Like the male parent, the plants were stoloniferous with rooting at the vegetative nodes. Inflorescences occurred in the leaf axils and petals were pink, like those of the maternal parent.

### Cytogenetic analysis and fertility of the hybrids

Two hybrid plants (440–1, 1854) were diploid (2n=16, Table 2, Figure 1g). Two others with tiny, indehiscent anthers and <1% stainable pollen were not studied further. Reciprocal GISH on somatic chromosomes of hybrid 440–1 confirmed that half the chromosomes were derived from *T. pallescens* (Figure 1g, h) and the remaining half from *T. occidentale* (Figure 1i). There was evidence of nucleolar dominance and GISH showed that the *T. pallescens*-derived NOR was decondensed and the *T. occidentale* NOR was condensed (Figure 1g–i).

**Table 2:** **Somatic chromosome number, meiotic configurations and pollen stainability in F**_**1,**_**F**_**2**_**and OP plants**

**Genotype**	**Somatic chromosome number**	**Number of PMC**	**Meiotic configurations**				**Anaphase I disjunction**	**Pollen stainability**
			**I**	**II**	**III**	**IV**		
			$\bar{\text{x}}$**(range)**	$\bar{\text{x}}$**(range)**	$\bar{\text{x}}$**(range)**	$\bar{\text{x}}$**(range)**		
440–1	2n=2x=16	>300	0 (0)	8 (0)	0 (0)	0 (0)	8–8	5%
88–01	2n=4x=32	80	0 (0)	8 (6–10)	0 (0)	4 (3–5)	16–16	78%
440–1 OP-3	2n=4x=32	70	0 (0)	8 (6–14)	0 (0)	4 (1–6)	16–16	18%
440–1 OP-9	2n=4x=32	55	0 (0)	9 (8–14)	0 (0)	3.5 (1–4)	16–16	27%
440–1 OP- 55	2n=4x=32	63	0 (0)	8.2 (6–12)	0 (0)	3.9 (2–5)	16–16	53%
440–1 OP-88	2n=4x+1=33	13	2.2 (1–5)	8.1 (2–14)	0.85 (0–2)	3.0 (0–5)	1617	43%
440–1 OP-153	2n=3x=24	43	2.0 (1–4)	2.6 (1–5)	5.6 (4–7)	0 (0)	12–12*	11%
F_2_ (2x)^†^	2n=2x=16	475	0 (0)	8 (0)	0 (0)	0 (0)	88	

* two cells showed 1113 disjunction and approximately 10 showed lagging chromosomes at anaphase I.

seven verified 2x plants produced from open pollination of 440–1. Two cells from one plant had 7 II and 2 I.

Meiotic analyses of hybrid 440–1 on a sample of >300 pollen mother cells (PMCs) showed all had eight bivalents (Table 2, Figure 1j) and 88 disjunction at anaphase I. GISH on PMCs of the hybrid (Figure 1k) confirmed homoeologous chromosome pairing as evidenced by a bivalent with 18S rDNA signals from both species and by the differential painting of other bivalents. Despite this apparent regularity, scanning of large numbers of PMCs revealed occasional multivalent formation and some unequal chromosome distribution in tetrads.

Both male and female fertility were low. As a guide to pollen fertility, the frequency of mature pollen stained was 5% in hybrid 440–1 (Table 2) and 1.52.0% in hybrid 1854. On the assumption of self-fertility (as for the female parent), more than 100 heads of hybrid 440–1 were self-pollinated but no pods or seeds developed. Subsequently, 20 cloned ramets of hybrid 440–1 (lacking leaf markings), were placed in an outdoor nursery alongside plants of *T. repens* (with co-dominant leaf markings) and the diploid parent species. Prolific flowering of 440–1 occurred over an extended period and hundreds of inflorescences were harvested. Open-pollinated (OP) seed-set was very low (less than one per 100 florets) but more than 200 OP seeds were obtained. Similarly, six ramets of hybrid 1854 were open-pollinated and about 35 seeds were obtained.

### Analysis of open-pollinated progeny

From hybrid 440–1, 184 seeds were imbibed and these produced 79 well-established, putative hybrid progeny plants. Of the remainder, 53 failed to germinate and 52 died as seedlings. Forty-four well-established plants were verified by DNA sequence analysis to be carrying *T. pallescens* chloroplasts from the maternal 440–1 plant. The others were either not tested (25) or were contaminants (10). More than half (26) of the verified plants resembled white clover in phenotype and/or carried leaf marks that indicated that the pollen parent was white clover (Table 3). Nineteen of these were tested and 18 were confirmed as tetraploid, i.e. derived by union of an unreduced (2n) egg from 440–1 with normal 2x pollen from *T. repens*. Such plants would be expected to have three satellite (NOR-bearing) chromosomes two from the 440–1 hybrid 2n egg and one from a normal 2x *T. repens* male gamete. This was checked for five plants and confirmed in all cases. One of the tetraploid plants with three NOR-chromosomes carried an additional chromosome (2n=4x+1=33). The last of the 19 plants, resembling a slender form of white clover, was verified as triploid (2n=3x=24). The presence of leaf markings and only two satellite chromosomes indicated that this triploid had arisen from a fusion between a haploid female gamete from 440–1 and a normal 2x white clover male gamete. The other 18 verified plants from 440–1 resembled diploid *T. pallescens* or *T. occidentale*. Chromosome counts or flow cytometry have confirmed all 12 tested plants to be diploid (Table 3). Open pollination of the second F_1_ (1854) produced 30 progeny plants (verified as above), three of which survived to flowering. Two were confirmed as tetraploid and the third died before it could be tested.

**Table 3:** **Characteristics of 440–1 OP progeny with maternity confirmed by presence of*T. pallescens trnL*intron**

**Appearance**	**Ploidy**	**No plants**	With leaf mark*	**No leaf mark**	**Pollen stainability (%)**				
					**ND**	**0–9**	**10–39**	**40–59**	**60+**
White clover-like	4x	19	16	3	4	1	5	8	1
	3x	1	1	−	−	−	1	−	−
	ND	6	6	−	1	1	3	1	0
Diploid-like	2x	12	2	10	6	2	3	1	0
	ND	6	1	5	3	2	1	0	0

* 4x plants carried leaf marks conditioned by V and R alleles from white clover [15]. Leaf-marked 2x plants resembled, and carried an R allele from, *T. occidentale.*

One 4x plant carried an additional chromosome.

ND (not determined).

The majority of the (*T. pallescens* x *T. occidentale*) x *T. repens* tetraploids were morphologically identical to white clover but showed lower male and female fertility and higher self-compatibility. A few plants showed developmental abnormalities such as small, wrinkled leaves and/or weak chlorophyll development in young leaves. Pollen stainabilities ranged from 072% (Table 3, control *T. repens* 98100%). Eleven verified tetraploid plants were self-pollinated and all except two produced seeds (433 per inflorescence compared with 0.6 per inflorescence for a white clover control), indicating self-compatibility (Table 4). The same plants were backcrossed as females to *T. repens* and all except the two apparently female-sterile plants produced 241 seeds per inflorescence, a marked reduction compared with over 100 seeds for control white clover (Table 5).

**Table 4:** Results of selfing tetraploid 440–1 OP progeny plants

**Plant selfed**	**No heads**	**No seeds**	**Seeds/head**
440–1 OP-3	2	19	9
440–1 OP-8	5	89	18
440–1 OP-9	5	88	18
440–1 OP-21	5	95	19
440–1 OP-57	4	35	9
440–1 OP-70	2	26	13
440–1 OP-72	3	100	33
440–1 OP-76	2	8	4
440–1 OP-88*	5	102	20
440–1 OP-90	4	0	0
440–1 OP-110	4	0	0
*T. repens*	5	3	0.6
440–1 OP-3 self-5	4	27	6
440–1 OP-3 self-12	5	3+7 small	2 small

* 2n=33 (carrying an additional chromosome).

**Table 5:** Seed-set from backcrossing verified tetraploid progeny plants of 440–1 to white clover (WC)

**Female**	**WC male**	**No. heads**	**No. seeds**	**Seeds/head**
440–1 OP-3	(PxB)-17	4	64	16
440–1 OP-8	C21557-1	1	22	22
440–1 OP-8	Will-2	3	53	18
440–1 OP-9	(PxB)-5	1 damaged	2	2
440–1 OP-21	(PxB)-5	3	39	13
440–1 OP-57	(PxB)-5	3	32	11
440–1 OP-70	C21557-1	2	5	3
440–1 OP-72	C21557-1	3	68	23
440–1 OP-76	C21557-1	2	7	4
440–1 OP-88	(PxB)-5	3	122	41
440–1 OP-90	C21557-1	2	2	1
440–1 OP-110	C21557-1	3	0	0
WC control	(PxB)-5	1	105	105
440–1 OP-3 self-5	(PxB)-5	3	10+3 small	3–4
440–1 OP-3 self-12	(PxB)-5	3	14+7 small	4–7

Four tetraploid plants and one triploid were analyzed for meiotic chromosome pairing (Table 2). All tetraploids averaged 34 quadrivalent associations per cell. One (440–1 OP-88) had an additional chromosome, and showed 15 univalents and 02 trivalents per cell at metaphase I. The triploid plant (440–1 OP-153) showed a predominance of trivalent chromosome associations and low numbers of univalents and bivalents (Table 2). The selfed seed from two tetraploid OP plants was grown. In one case (440–1 OP-3 selfed), 19 seeds gave 17 plants, of which 12 were robust and white clover-like, three were slightly less robust and two were small with abnormal leaf morphology and/or pale green leaf sectors. The second plant (440–1 OP-4) was abnormal and, when six selfed seeds were grown, gave two strong, one medium and three very weak plants. When two 440–1 OP-3 selfed progeny plants were further selfed and backcrossed to white clover, the seed-sets were low, indicating reduced fertility (Tables4 and 5).

### An artificial allotetraploid (synthetic white clover)

The chromosomes of hybrid 440–1 were colchicine-doubled to produce an amphidiploid (8801). This plant showed an improved pollen stainability of 78%, was self-compatible and freely set seeds in reciprocal crosses with white clover (Table 6). Self-pollination of 8801 produced about 4 seeds/head and led to about 50% fully developed seeds and 50% shrunken seeds and unfilled testas. Fourteen selfed-seeds were set for germination, 11 germinated, and 9 grew to mature plants. Cross-pollination of 880–1 with white clover plants gave some seed-sets per head of over 40 (Table 6). Thus, the raw synthetic hybrid was reasonably freely inter-fertile with white clover. Analysis of the progeny plants from these crosses showed reduced survival, pollen stainability and seed-set relative to white clover controls (Table 7), although some synthetic derivatives had pollen fertility approaching white clover plants. One of the self-progeny plants (88–01 self-5), was self-fertile and averaged 32 and 82 seeds per head when crossed as male with two white clover plants, including one individual with 127 seeds – the same as the white clover control (Table 6) – indicating that a selfed derivative of the synthetic allotetraploid was highly inter-fertile with white clover.

**Table 6:** Seed-set following self-pollination and reciprocal crosses with white clover for hybrid 8801 and a selfed derivative

**Female x Male**	**Heads**	**Seeds**	**Seeds/head**
4x hybrid self pollinations			
88–01 self	8	32	4
88–01 self-5 self	8	155	19
4x hybrid x white clover			
88–01 x Crusader-29	9	40	4
88–01 x Kopcru-1	5	41	8
White clover x 4x hybrid			
Kopu II-2 x 880-1	2	88	44
Kopcru-1 x 880-1	12	384	32
C 64524 x 880-1	2	90	45
C 115194 x 880-1	3	55	18
Kopu II-2 x 880–1 self-5	2	164	82
Kopcru-1 x 880–1 self-5	4	130	32
White clover control			
Kopu II-2 x Crusader-29	1	120	120

**Table 7:** Viability of progeny from crosses between white clover plants and hybrid 880-1

**Female x Male**	**No seeds**	**No plants**	**No plants flowering**	**PS%** **(range)**	**Seeds/head (range)**
88–01 x Crusader-29	12	12	12	3–85	4–50
88–01 x Kopcru-1	10	10	10	0–73	0–19
Kopu II-2 x 880-1	11	6	5	22–62	9–29
Kopcru-1 x 880-1	12	12	12	21–63	1–21
C6452-4 x 880-1	12	11	11	24–61	0–29
C11519-4 x 880-1	12	10	7	34–78	1–34
Kopu II-2 x Crusader-29	12	12	12	98–100	230–420

Seeds/head data were based on means of a minimum of three open-pollinated heads per plant at a single harvest. PS%=% stained pollen.

### Attempts to obtain a spontaneous allotetraploid

The confirmation of tetraploid progeny following open-pollination of 440–1 indicated the functioning of 2n female gametes in this diploid hybrid. On the assumption that 2n male gametes might also be functional, several attempts were made to form an allotetraploid by self pollination. However, this was unsuccessful despite more than 1,000 hand pollinations with large amounts of pollen, and bee pollinations of hundreds of inflorescences in an insect cage. Hand pollinations of 440–1 with known diploid pollen sources (88–01 and *T. repens*) also failed to produce seeds, suggesting that the frequency of female unreduced gametes was too low to enable detection from these controlled crosses.

## Discussion

The present evidence validates the hypothesis that white clover, *T. repens*, arose following the hybridization of progenitor taxa very similar to modern *T. pallescens* and *T. occidentale* to form a partially fertile diploid hybrid(s). In the present experimental open pollination situation, unreduced (2n) gametes from the hybrids readily combined with natural (2x) gametes from white clover to produce hybrids that closely resembled white clover. We hypothesize that the original F_1_ hybrid(s) produced unreduced gametes, leading to a transgressive allotetraploid form. Such allotetraploids frequently exhibit markedly better fertility and broader adaptive capacity through polyploidy, heterozygosity and genomic plasticity [16]. Thus, *T. repens*, a widely adapted aggressive weedy species (with agronomic value as well) arose from two species, the modern forms of which are now comparatively rare and narrowly adapted. The primary allotetraploid probably had two pairs of NOR-carrying chromosomes. Post-hybridization genomic changes have reduced the NORs to one pair in modern white clover populations.

Analyses presented here provide evidence that *T. pallescens* and *T. occidentale* are the closest known modern relatives of the ancestral parents of white clover. It was shown that the chloroplast *trnL* intron DNA sequences of two populations of *T. pallescens* were identical with white clover, and another three populations were more similar than any other known species to those of *T. repens*. In addition, reciprocal GISH experiments demonstrated that half of the chromosomes of *T. repens* hybridized strongly with genomic DNA of *T. pallescens* and the other half with *T. occidentale* genomic DNA*.* Furthermore, plants from *T. pallescens* (AZ1895) could be crossed with *T. occidentale* to produce diploid hybrids that were inter-fertile with white clover via unreduced gametes. Thus these modern populations were close enough to the ancestors to be able to replace them in both GISH and in the production of synthetic hybrids.

During glacial episodes in Europe, alpine species such as *T. pallescens* were forced into low altitude refuges [17,18]. Such refuges included coastal regions of W Portugal and Spain, where *T. occidentale* currently occurs, as well as regions further east (e.g. the Balkans) where *T. repens* var. *biasolettii* and var. *macrorrhizum (*forms similar to *T. occidentale*[19]) occur. Hybridization might, therefore, have taken place in such a refuge during a glacial period. The small divergences in DNA sequences between extant *T. pallescens* populations from the Pyrenees, the Austrian Alps and N Greece are consistent with their separate evolution as they subsequently regressed to different alpine regions as the climate warmed after successive glaciations. The largest DNA sequence differences in the chloroplast *trn*L intron between white clover and extant *T. pallescens* populations can be explained by single insertion/deletion changes. Similarly, the largest differences found to-date between *T. occidentale* and *T. repens* ITS sequences were single bp differences. Such singular changes cannot be dated, and so a molecular clock cannot yet be applied. Their singular nature suggests that they could be recent, possibly occurring during or since the period of complex temperature fluctuations spanning the last two major glaciations 130,000-13,000years ago [20].

Although there has been no comprehensive study of chloroplast inheritance patterns in *Trifolium,* a limited study of several species, including white clover, indicated that maternal inheritance occurred without exception [21]. In the present study, we found that maternal inheritance of *T. pallescens* chloroplasts occurred and, furthermore, used this to verify the authenticity of the progeny obtained from the F_1_ hybrids. Thus it is likely that *T. pallescens* was the maternal ancestor.

It is expected that some genomic divergence could have occurred since hybridization not only within the parent species, but also in the sub-genomes within white clover populations. The extent of such divergences would reflect the length of time as well as the effects of habitat pressures and natural selection since hybridization. In this context, the identity, and close similarity, of chloroplast DNA sequences, and the GISH and plant hybridization results are compelling evidence in favor of *T. pallescens* as the maternal ancestor. Any alternative female ancestor must have been so close to *T. pallescens* as to have strong affinities in the dispersed repeat DNA responsible for GISH genomic differentiation. It must also have been so close that the modern *T. pallescens* genome can substitute functionally for it in artificial hybrids. However, the need for embryo rescue to achieve the crosses suggests some difference in reproductive biology from ancestral forms. In the same way, similarities of *T. occidentale* nuclear DNA sequences with those of *T. repens* provide strong evidence that *T. occidentale* was the other (and probably the male) ancestor.

*T. pallescens* has evolved into divergent, widely separated sub-alpine and alpine populations in the Pyrenees and Massif Centrale and across the Alps of C and S Europe to Greece, Romania and Bulgaria. We have shown that populations from the Pyrenees, Austrian Alps and Greece differed in *trnL* intron DNA sequences. Apparently different forms of *T. pallescens* have developed in isolation in different alpine islands in Europe, possibly as the populations retreated to alpine habitats with climate warming, as discussed above. Even populations of *T. pallescens* in adjacent valleys in the Tyrol show some genetic differentiation [10]. It would therefore not be surprising if populations as far apart as Spain and Greece showed considerable differentiation. The finding [11] that nuclear gene SNPs differentiated the *T. pallescens* (AZ1895) genome from both sub-genomes of white clover may be consistent with the finding of genomic variation among *T. pallescens* populations.

While it is significant that at least two specimens (from Greece) have been found with identical *trnL* intron sequences to that of white clover, it is likely that a wider sampling of *T pallescens* genomic variation would reveal more about the distribution of the ancestral populations and the region of origin of white clover. The great geographic distance between extant *T. occidentale* and the Greek *T. pallescens* populations is inconsistent with the hypothesis that they formed hybrids. Further sampling might reveal *T. pallescens* populations with identical cpDNA sequences to white clover in W Europe. Alternatively, *T. occidentale* might previously have had a wider distribution. Clover forms from eastern Europe, Turkey and Iran with hairy petioles and pedicels similar to *T. occidentale* are *T. repens* var. *biasolettii* and var. *macrorrhizum*[19], which could represent relict eastern forms. However, our DNA sequence analyses to-date (one specimen of each) suggest that they are forms of white clover.

White clover and its wild relatives occur as a species complex with close affinities in DNA sequences, chromosome pairing and crossability, indicating recent and rapid speciation and distribution across Europe, W Asia and N Africa [22]. The complex includes several other species in section *Trifoliastrum*[8,23]: *T. nigrescensT. ambiguumT. uniflorum, T. isthmocarpum* and *T. thalii*[24]. Although hybridization between *T. pallescens* and *T. occidentale* appears to have been the main factor in the origin of white clover, some introgression from other species in the complex may also have occurred. One gene that may have introgressed in this manner is *Li*, conditioning the production of linamarase, an enzyme that degrades cyanogenic glucosides, and one that is very common in white clover [25]. So far *Li* has not been found in either *T. pallescens* or *T. occidentale*, although the latter is polymorphic for production of cyanogenic glucosides [26]. The only close relative known to carry *Li* is *T. nigrescens*[27,28] which can hybridize with both *T. repens* and *T. occidentale*[7], providing possible alternative routes for introgression.

Both ancestral species are predominantly self-compatible but have given rise to a self-incompatible species with a well characterized gametophytic oppositional *S* allele incompatibility system [15]. Therefore the origin of the white clover *S* locus should be addressed. One possibility is that it came from the ancestral *T. occidentale* populations. Extant populations of *T. occidentale* from a confined region of NW Spain are self-incompatible [12] and so the *S* locus could have been introduced directly or indirectly from the ancestral source. Alternatively, as *T. nigrescens* also has a well defined *S* allele incompatibility system [29], it could have come by introgression from that species as suggested for *Li*.

A property of new allopolyploids, contributing to their evolutionary success, is genomic plasticity [16,30]. This plasticity provides the ability to withstand large, rapid genomic changes including diploidization, and leads to the development of new phenotypes and adaptations. Such changes have occurred during the evolution of white clover. One of the most obvious has been diploidization of the NOR regions [1]. GISH confirmed this and showed the apparently complete loss of the *T. pallescens* NOR regions (Figure 1c–e). Another post-polyploidization change is that all 32 centromeres of *T. repens* have large blocks of TrR350 tandem repeat DNA whereas *T. pallescens* has TrR350 on only 4 chromosome pairs [14]. Concerted evolution in the allotetraploid genome has apparently led to the spread of this satellite DNA to all centromeres.

The strong chromosome pairing affinities in hybrid 440–1 between the ancestral species implies that these diploid species evolved from a common ancestor recently enough that chromosome pairing affinity is retained, despite substantial evolutionary changes in adaptations, phenotypes and other genomic properties. By contrast, the existence of near-perfect homologous bivalent pairing in *T. repens*[15] suggests that the ancestral genomic pairing affinities may have been brought under genetic control or otherwise suppressed since the formation of white clover.

The use of *T. repens* as a pollen source for 440–1 effectively provided a genetic sieve for unreduced female gametes from the interspecific hybrid. More than half (26/44) of the verified, fully functional, female gametes sampled here from hybrid 440–1 were unreduced. The open pollination set-up enabled tens of thousands of hybrid florets with hundreds of thousands of egg cells to be fertilized with a mixture of n and 2n pollen. The finding of as few as 26 functional unreduced female gametes among hundreds of thousands indicated that the frequency of such gametes was very low. This also provided an insight as to why no spontaneous allotetraploids were found from either controlled crosses or open-pollination.

Transgressive gene interactions provide phenotypic innovations during hybrid speciation [31-33]. The transgressive adaptation of white clover probably arose from multiple genetic and epigenetic interactions involving complementarities and epistatic interactions between the alpine adaptation of one parent and the coastal adaptation of the other parent. An example may be the combining of the stoloniferous habit of *T. occidentale* with the ability of *T. pallescens* to grow in relatively infertile non-saline inland soils. This would have been accompanied by the ecological opportunity for a stoloniferous clonal herb to colonize post-glacial grasslands grazed by expanding populations of animals. Allopolyploidy would have served to stabilize the transgressive genetic and epigenetic interactions, achieve reproductive isolation, enhance fertility and probably also would have provided enhanced vegetative vigor. The raw diploid and artificial tetraploid hybrids obtained here will enable breeders to introgress traits from modern *T. pallescens* and *T. occidentale* into white clover. They may also provide a valuable resource for the study of transgressive genetic and epigenetic interactions in adaptive radiation and speciation.

### Implications for clover breeding

Based on the above evidence, it is apparent that the sub-genomes in white clover (designated P^r^P^r^O^r^O^r^) differ from those in contemporary *T. pallescens* (P^p^P^p^) and *T. occidentale* (O^o^O^o^). There is evidence for multivalent chromosome configurations in synthetic hybrids (Table 2). Hybrids between synthetic and natural forms of white clover should be P^p^P^r^O^o^O^r^, and chromosome pairing would enable introgression of new alleles from the ancestral species into white clover. Such introgression could provide genes for new traits (e.g. drought tolerance from *T. occidentale*) as well as in-built heterotic interactions arising from new inter-sub-genomic heterozygosity. Additionally, other genomic reconfigurations such as those described for tomato interspecific introgression lines [33] and synthetic *Brassica napus*[34,35] may provide beneficial transgressive genomic interactions. Synthetic forms of white clover could therefore be used in breeding to improve vigor and broaden the adaptation of new cultivars, e.g. to semi-arid and saline environments.

## Conclusions

Experimental evidence obtained from DNA sequence analyses, molecular cytogenetics, interspecific hybridization and breeding research is consistent with the hypothesis that the diploid alpine species *T. pallescens* hybridized with the diploid coastal species *T. occidentale* to form tetraploid *T. repens*. The coming together of these two species with very narrow but different adaptations, along with allotetraploidy, has produced a transgressive hybrid with a broad adaptive range. F_1_ interspecific hybrids generated by embryo rescue are inter-fertile with white clover and function as synthetic white clovers, a valuable new resource for the re-incorporation of ancestral genomes into modern white clover for future plant breeding.

## Methods

### Plant material

Living plants used in this study were derived from seeds from the Margot Forde Forage Germplasm Centre, AgResearch Grassland Research Centre, Palmerston North, New Zealand. *T. pallescens* AZ1895 was of undesignated origin, while AZ4837 was collected from above Nuria, at 2,100m altitude in the Spanish Pyrenees and AZ4856 was from a similar altitude in the Rotmoos Valley, Tyrol Alps, Austria. *T. pallescens* plants from N Greece were analyzed from herbarium samples 16156, 19230, registered for the Flora Hellenica Database. *T. occidentale* accessions OCD 1162 (Faro de Cabo Villano), OCD 1163 (Camarinas), OCD 1166 (Beo Peninsular) and OCD 1168 (Punta Frouxeira beach) were from sea level on the coast of NW Spain.

### Wide hybridization and embryo rescue

All *T. pallescens* plants were self-fertile and so emasculation and hand-pollination were required. Emasculated florets were pollinated several times over two days and embryos were removed after nine days and placed on a shoot proliferating medium CR7 [36]. Developing shoots were subsequently transferred to a root initiation medium CR5 [36] before finally being planted into potting mix in the greenhouse. Putative hybrids were grown to maturity and verified by DNA sequence analysis of ITS and chloroplast *trn*L regions using methods described in [8]. At flowering, self-pollination and cross-pollination with sibs and parent species were carried out by hand in an insect-free greenhouse. Subsequently, the plants were placed outside for a full seasonal cycle to open-pollinate with the parental species and white clover. Seed-set under these conditions provided an indication of female fertility. Male fertility was estimated by extracting mature pollen onto a microscope slide, staining with 2% acetocarmine, and counting the number of full-sized, fully stained grains in a minimum sample of 300 grains (200x magnification). The white clover plants used in this study carried the co-dominant leaf markings ‘white V’ and/or ‘red leaf’, conditioned by alleles at the V and R loci, respectively [15]. Expression of these alleles in progeny derived from using these plants as male parents provided evidence of paternity. DNA sequence analysis of the chloroplast *trn*L regions of the progeny were used to verify maternity.

### Chromosome doubling and subsequent crossing with white clover

Colchicine-doubling of 440–1 axillary meristems was carried out by a previously described method [37] using 0.07% colchicine for 60 hours in the dark at 4C. Chromosome doubled plants were identified initially by increased pollen stainability and verified by morphology of dry pollen grains and chromosome counts. All progeny were verified using leaf marks and/or ITS and chloroplast DNA sequences.

### DNA analysis and molecular cytogenetics

Somatic chromosome preparations were obtained from actively growing root tips after hydrolyzing with HCl and squashing in acetocarmine or using a flame-drying technique after enzymatic maceration as described earlier [1]. Meiotic chromosome preparations were obtained by squashing pollen mother cells from young floral buds either after enzymatic maceration or after staining with alcoholic hydrochloric acid carmine [38]. Somatic and meiotic preparations obtained after enzymatic maceration were used for GISH-FISH experiments.

Total DNA was prepared from fresh leaf samples using the method of Lefort and Douglas [39] with modifications as described in [8]. Total DNA was prepared from herbarium-derived leaf samples using the DNeasy Plant Mini Kit (QIAGEN, Germany) using the manufacturers protocol, except that the elution buffer was pre-heated to 65 °C. The nuclear ITS and chloroplast *trnL* intron regions were amplified and sequenced as described in [8]. The DNA probes for GISH-FISH experiments were: genomic DNA of *T. occidentale* and *T. pallescens*; pTr5S (GenBank AF072692), a 596bp fragment from *T. repens* representing part of the 5S rDNA gene family; and pTr18S (GenBank AF071069), a 1.8kb fragment from *T. repens* containing almost an entire 18S rDNA sequence. Genomic DNA, isolated from *T. pallescens* and *T. occidentale*, and the two types of rDNA were individually labeled with fluorochrome-labeled nucleotides Cy3-dCTP or FluorX-dCTP (GE Healthcare) by nick translation according to the manufacturers specifications. Procedures for *in situ* hybridization, post-hybridization stringent washing and DAPI counterstaining of chromosomes have been described [1,30]. Somatic chromosomes from *T. repens* and somatic as well as meiotic chromosomes from hybrid 440–1 were subjected to GISH-FISH using Fluor-X-labeled genomic DNA of *T. pallescens* and Cy3-labeled 18S rDNA. After recording the images, the same somatic preparations from *T. repens* and hybrid 440–1 were re-probed for reciprocal GISH-FISH using Cy3-labeled genomic DNA of *T. occidentale* and Fluor-X-labeled 5S rDNA. The former GISH-FISH experiment included unlabeled *T. occidentale* genomic DNA while the latter experiment included *T. pallescens* genomic DNA as blocking DNAs. Slides were examined under a Nikon Microphot-SA epifluorscence microscope. The images were captured using an AxioCam MRm CCD camera (Carl Zeiss GmbH, Germany) attached to the microscope and processed with ISIS imaging software (MetaSystems GmbH, Germany). Individual photographs were composed on plates using Adobe Photoshop software.

## Authors contributions

All authors were involved in designing and planning the research. NWE carried out DNA work, analyzed DNA sequences and supported molecular cytogenetics, HAA performed and analyzed molecular cytogenetics experiments, IMV made the hybrids, carried out embryo rescue and generated derived progeny, SWH provided cytological results, WMW derived and analyzed progeny and wrote the paper. All authors read and approved the final manuscript.
